# Microbiota and their metabolites potentiate cancer immunotherapy: Therapeutic target or resource for small molecule drug discovery?

**DOI:** 10.3389/fphar.2022.1091124

**Published:** 2022-12-15

**Authors:** Peixin Du, Jing Jing, Xiujing He

**Affiliations:** Laboratory of Integrative Medicine, Clinical Research Center for Breast, State Key Laboratory of Biotherapy, West China Hospital, Sichuan University, and Collaborative Innovation Center, Chengdu, Sichuan, China

**Keywords:** microbiota, microbial metabolites, small molecule drugs, immune checkpoint inhibitors, immunotherapy response

## Abstract

Increasing evidence has proved that microbiota is not only the target of small molecule drugs but also an underexplored resource for developing small molecule drugs. Meanwhile, microbiota as a critical modulator of the immune system impacts the efficacy and toxicity of cancer immunotherapy. Harnessing microbiota or developing microbiota-derived medications provide novel therapeutic strategies to overcome resistance to cancer immunotherapy and immune-related adverse events (irAEs). In this review, we elucidate how microbiota and their metabolites impact anti-tumor immunity and immunotherapy efficacy and highlight the potential of microbiota and their metabolites as a resource for small molecule drug discovery. We further overview the current landscape of clinical trials evaluating the potential effect of microbiota and their metabolites on immunotherapy outcomes, presenting future trends in the field of microbiota-based therapies. Microbiota-based therapies are promising therapeutic options to promote therapeutic efficacy and diminish the toxicity of immunotherapy.

## Introduction

Increasing evidence suggests that microbiota composition is not only associated with several human diseases such as autoimmune disease and cancer (Kostic et al., 2013; Scher et al., 2013; Huttenhower et al., 2014; Matson et al., 2021; Connell et al., 2022), but is also responsible for therapeutic efficacy (Allen-Vercoe and Coburn, 2020; Finlay et al., 2020; Huang et al., 2020; Ansaldo and Belkaid, 2021; Pham et al., 2021; Singh et al., 2021), drug resistance (Baruch et al., 2021a; Goc and Sonnenberg, 2022), and adverse effects of multiple therapies (Pham et al., 2021; Seton-Rogers, 2021). Small molecule drugs can be used to ameliorate the altered microbiota composition of cancer patients (Vezza et al., 2019; Huang et al., 2022). More crucially, emerging evidence highlights that microbiota is an invaluable resource for discovering small molecule drugs and microbiota-derived medications have wide applications in anticancer therapy.

**FIGURE 1:**
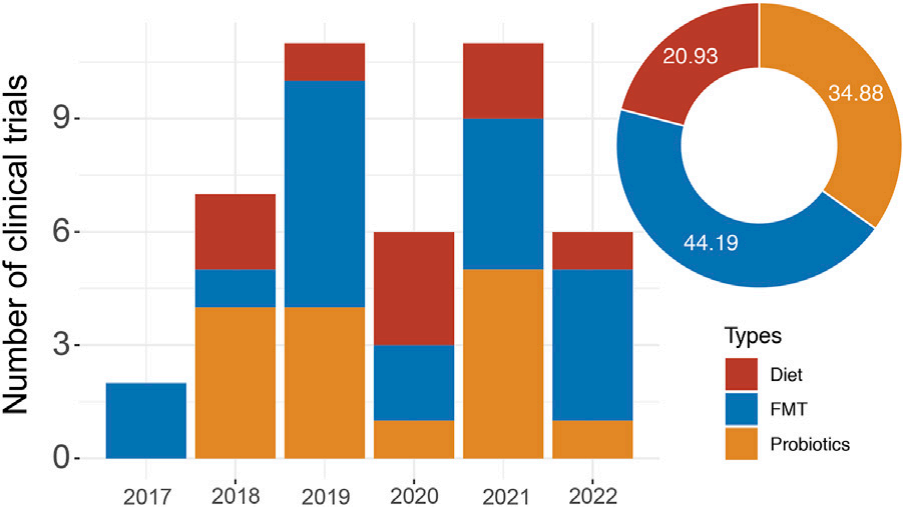
The clinical trial landscape of immunotherapy in combination with microbiota-based therapy. The graph shows the number of combination trials starting each year since 2017. The pie chart shows the proportion of different microbiota-based therapies.

Microbiota affects the host in several ways, including the release of metabolic products, cellular components, and secreted proteins that can activate various host receptors to modulate immune responses. Especially, microbiota-derived metabolites have obvious safety advantages over microbiota intervention, without the risk of systemic infection. Microbial metabolites, such as short-chain fatty acids (SCFAs), bile acid, lactic acid, spermidine, indole, and retinoic acid, have been demonstrated to link the intestinal microbiome to systemic immunity (Levy et al., 2017), indicating a future potential perspective in the treatment of tumors (Zhou and Fang, 2018). Furthermore, microbiota and their metabolites also affect the efficacy and toxicity of immunotherapy by modulating host immune responses *via* different regulatory mechanisms (Inamura, 2020; Zhou et al., 2021). Hence, it is essential to exploit the microbiota and their metabolites to develop novel therapeutic strategies as well as to identify druggable targets that can assist cancer immunotherapy. Here, we highlight recently gained insights into the immunomodulatory functions of microbiota and their metabolites and further demonstrate the unique impacts of microbiota-based therapies on immunotherapy efficacy in patients with cancer, by providing evidence from clinical trials (Figure 1).

## Microbiota is an underexplored resource for small molecule drug discovery

A significant metabolic function of the intestinal microbiome is the anaerobic fermentation of ingested dietary fiber and mucosal glycans to produce SCFAs, such as acetate, propionate, and butyrate (Kim et al., 2017). SCFAs have extensive effects on the physiology of the host, especially immunological regulation (Buck et al., 2017). By inhibiting histone deacetylase or combining G-protein-coupled receptors, SCFAs regulate the release of cytokines (Li et al., 2018), as well as the activity of B cells, T cells, Tregs, and innate immune cells (Trompette et al., 2018; Kim, 2021). Specifically, butyrate can increase the clearance of activated T cells by upregulating the Fas cell surface death receptor (Furusawa et al., 2013). In a clinical study of solid tumor patients treated with anti-CTLA-4 antibody, low levels of serum butyrate and propionate were observed to be associated with prolonged progression-free survival (PFS) in the pooled cohort (*n* = 85), and there was a correlation between gut bacteria and systemic SCFA concentrations (Coutzac et al., 2020). The findings from the other two clinical studies, however, are in contrast with those shown above. Higher fecal SCFA concentrations were substantially related to prolonged PFS in a study of 52 patients with solid tumors under anti-PD-1 treatment (Nomura et al., 2020). Another study indicated that fecal SCFAs, particularly propionate, were associated with improved long-term responses to ICIs in non-small cell lung cancer (NSCLC) patients treated with anti-PD-1 antibody (Botticelli et al., 2020).

Bile acids (BAs) are another type of metabolite biotransformed by microbiota that exert immunomodulatory properties in the intestine of the host (Campbell et al., 2020; Li et al., 2021). Crosstalk between intestinal microbiota, BAs, and the host influences immune functions, metabolic phenotypes, and risk factors for various cancers (Schroeder and Bäckhed, 2016; Sun et al., 2021). Several intestinal strains of the genus Clostridium have been discovered to produce secondary BAs such as deoxycholic acid (DCA) and lithocholic acid (LCA) by removing the 7α/β-hydroxy group from primary BAs (Ridlon et al., 2016). One of the BAs called isodeoxycholic acid produced by gut bacteria through the epimerization of DCA (Devlin and Fischbach, 2015) increases the production of peripherally generated Treg cells by suppressing the immunostimulatory capacity of dendritic cells (Campbell et al., 2020). Moreover, a preclinical study showed that the LCA derivative 3-oxoLCA inhibited T_H_17 cell differentiation, whereas another derivative of LCA called isoalloLCA boosted Treg cell expansion (Hang et al., 2019).

As a necessary amino acid for humans, tryptophan and its metabolites are known to be bioactive substances that can affect immune cell differentiation by acting as arylhydrocarbon receptor (AhR) ligands (Sun et al., 2020) and thus have significant effects on the regulation of the immune system and the development of cancer. Most of the dietary tryptophan is absorbed in the small intestine, while a minor amount reaches the colon and is metabolized by the intestinal microbiome (Cryan et al., 2019). Kynurenine produced from tryptophan was the most remarkably elevated serum metabolite in response to anti-PD-1 antibody, and an increased serum kynurenine/tryptophan ratio was related to shorter overall survival in melanoma and renal cell carcinoma (RCC) patients (Li et al., 2019). A clinical study that evaluated plasma tryptophan metabolites in 19 NSCLC patients treated with ICIs discovered that low levels of 3-hydrozyanthranilic acid were substantially correlated with prolonged median PFS (Karayama et al., 2021).

Inosine derived from gut microbes has also been demonstrated to be associated with ICI therapy responses. He et al. (2017) found that the gut microbiota regulated the concentration of inosine, which inhibited Th1/Th2 cell differentiation by combining with adenosine A2A receptor. Another study demonstrated that inosine improved anti-tumor responses of immunotherapy in mouse models of various cancer types (Mager et al., 2020). Furthermore, the combination of inosine and a PD-L1 inhibitor resulted in delayed tumor growth and prolonged survival time in a melanoma mouse model (Wang et al., 2020). However, some cancer cells compete with T cells for inosine as an alternative energy source when glucose is lacking, dampening the efficacy of inosine supplementation in combination with anti-PD-L1 therapy. Conversely, several microbial metabolites, such as polyamines and lipoteichoic acid, contribute to the shaping of the tumor-promoting microenvironment (Loo et al., 2017; Proietti et al., 2020). With a growing number of microbiota-derived mediators being discovered, those that cause favorable immune responses in the host are being turned into possible therapeutic medications.

## Microbiota and their metabolites can improve the benefit of immunotherapy

### Fecal microbiota transplantation (FMT)

One effective strategy for manipulating the gut microbiota is through FMT. During this process, fecal material from a carefully screened healthy donor is transferred to a recipient *via* colonoscopy, nasogastric tube, or prepared capsules. FMT has been used to treat several clinical indications, including *Clostridium difficile* infection, ulcerative colitis, and other gastrointestinal conditions. Recently, FMT has been investigated in conjunction with immunotherapy (especially checkpoint blockade therapy) as a possible treatment strategy.

In a phase I clinical trial (NCT03353402), researchers evaluated the capacity of responder-derived FMT to rescue the clinical efficacy of ICIs in metastatic melanoma patients who failed previous immunotherapy. The FMT from two donors was transferred to ten enrolled patients *via* colonoscopy, followed by stool-microbiota capsules before combined treatment with the anti-PD-1 antibody nivolumab. Interestingly, an objective response was observed in three patients who received FMT from the same donor, leading to an overall response rate of 30% (3/10). Notably, all five recipients in this donor group had favorable changes in immune parameters, manifesting as the upregulation of genes related to antigen presentation and innate immunity. Furthermore, this study confirmed that treatment with FMT could alter the gut microbiome and induce changes in the activation of anticancer immunity and the tumor microenvironment (TME) in recipients (Baruch et al., 2021b). Similar results have been found in other cohorts of patients with metastatic ICI-resistant melanoma (NCT03341143). The combination of FMT and anti-PD-1 antibody provided clinical benefit in 40% (6/15) of patients and induced rapid microbiota perturbation and TME reprogramming to overcome resistance to anti-PD-1 therapy (Davar et al., 2021). In an ongoing phase I trial (NCT03772899) designed to investigate the safety of FMT and immunotherapy combination, twenty patients with ICI-naive advanced melanoma received FMT *via* oral capsules, followed by anti-PD-1 treatment. Initial data demonstrated that the objective response rate (ORR) was 65%, as 13 out of 20 patients achieved CR or PR, with a clinical benefit rate of 75%. Moreover, parallel experiments in mice corroborated that FMT could contribute to the antitumor response and restore anti-PD-1 efficacy (Miller et al., 2022). The above findings showed that combining FMT and anti-PD-1 therapy can enhance antitumor immunity and potential clinical responses by rebuilding the gut microbiota and modifying the TME. More ongoing clinical studies focusing on the efficacy of FMT as an adjuvant of ICI treatments are currently underway across several malignancies, aiming at boosting the response rate to ICIs (Table 1).

**TABLE 1:** Clinical evidence linking gut microbiota and cancer immunotherapy.

Trial number	Cancer	Sample size	Cancer treatment	Types of microbial intervention	Microbial intervention	Status
NCT03353402	Advanced melanoma	40	Anti-PD-1	FMT	FMT from responders *via* colonoscopy and capsule	Unknown
NCT03341143	Advanced melanoma	18	Anti-PD-1	FMT	FMT from responders *via* colonoscopy	Active, not recruiting
NCT03637803	Advanced solid tumor	132	Anti-PD-1	Probiotics	MRx0518	Recruiting
NCT04056026	Metastatic mesothelioma	1	Anti-PD-1	FMT	FMT *via* colonoscopy	Completed
NCT03772899	Advanced melanoma	20	Anti-PD-1	FMT	FMT *via* colonoscopy	Active, not recruiting
NCT04163289	Advanced/metastatic RCC	20	Anti-CTLA-4 + anti-PD-1	FMT	FMT *via* capsule	Recruiting
NCT04130763	Gastrointestinal cancer	10	Anti-PD-1	FMT	FMT *via* capsule	Recruiting
NCT03829111	Advanced/metastatic RCC	30	Anti-CTLA-4 + anti-PD-1	Probiotics	CBM 588	Recruiting
NCT03817125	Advanced/metastatic melanoma	14	Anti-PD-1	Probiotics	SER-401	Completed
NCT03686202	Solid tumor	65	Anti-PD-1/PD-L1	Probiotics	MET-4	Recruiting
NCT03595683	Advanced melanoma	8	Anti-PD-1	Probiotics	EDP1503	Recruiting
NCT04009122	Metastatic NSCLC	280	—	Diet	IGEN-0206 (dietary nutritional product)	Recruiting
NCT04552418	Solid tumor	12	Anti-CTLA-4 + anti-PD-1	Diet	Potato starch	Recruiting
NCT04107168	Melanoma, Renal cancer, Lung cancer	1800	Anti-PD(L)1 ± anti-CTLA-4	—	—	Recruiting
NCT03819296	Cutaneous melanoma, Malignant genitourinary system neoplasm, Malignant solid neoplasm, Lung cancer	800	Immunotherapy	FMT	FMT	Recruiting
NCT04204434	Advanced solid tumor	150	Immunotherapy	—	—	Recruiting
NCT04579978	Advanced solid tumor	60	Immunotherapy	—	—	Recruiting
NCT04038619	Genitourinary malignancy	40	Immunotherapy	FMT	FMT *via* colonoscopy	Recruiting
NCT04758507	RCC	50	Immunotherapy	FMT	FMT	Recruiting
NCT04189679	NSCLC	60	Immunotherapy	—	—	Recruiting
NCT03775850	Multiple solid tumors (Colorectal, TNBC, NSCLC, Bladder cancer, RCC, Gastroesophageal cancer)	69	Anti-PD-1	Probiotics	EDP1503	Completed
NCT04116775	Prostate cancer	32	Anti-PD-1	FMT	FMT *via* colonoscopy	Recruiting
NCT04645680	Metastatic melanoma	42	Anti-PD-1	Diet	High fiber diet	Recruiting
NCT04167137	Metastatic solid tumor, Lymphoma	70	Anti-PD-1	Probiotics	SYNB1891	Recruiting
NCT04883762	Tumor	10	Anti-CTLA-4/PD-1/PDL-1	FMT	FMT *via* colonoscopy	Recruiting
NCT05273255	Tumor	30	Immunotherapy	FMT	FMT *via* colonoscopy	Recruiting
NCT04951583	Advanced NSCLC, melanoma	70	Anti-PD-1 ± anti-CTLA-4	FMT	FMT *via* capsule	Recruiting
NCT04711330	NSCLC	126	Anti-PD-L1	—	—	Not yet recruiting
NCT03688347	Lung cancer and other malignancies	44	Immunotherapy/chemotherapy	—	—	Completed
NCT04954885	Non-squamous NSCLC	150	Anti-PD-1	—	—	Recruiting
NCT03643289	Melanoma	450	Immunotherapy	—	—	Recruiting
NCT05220124	Bladder urothelial carcinoma	190	Immunotherapy	Probiotics	Live combined capsules (Bifidobacterium, Lactobacillus and Enterococcus)	Recruiting
NCT05251389	Melanoma	24	Anti-PD-1	FMT	FMT	Recruiting
NCT05286294	Melanoma, Head and neck squamous cell carcinoma, Cutaneous squamous cell carcinoma, Clear cell renal cell carcinoma	20	Anti-CTLA-4 ± anti-PD1/PD-L1	FMT	FMT	Recruiting
NCT04924374	Lung cancer	20	Anti-PD-1	FMT	Pooled fecal microbiota capsules	Recruiting
NCT04699721	NSCLC	40	Neoadjuvant chemotherapy + Anti-PD-1	Probiotics	BiFico (Bifidobacterium trifidum live powder)	Recruiting
NCT03700437	NSCLC	12	Chemo-immunotherapy (Anti-PD-1)	Diet	FMD	Completed
NCT04866810	Melanoma	80	Anti-LAG3 + anti-PD-1	Diet	High fiber, plant based diet	Recruiting
NCT05356182	Solid tumor	30	Anti-PD-1/PD-L1 ± anti-CTLA-4	Diet	Low-protein diet	Recruiting
NCT03709147	Advanced LKB1-inactive lung adenocarcinoma	64	Chemo-immunotherapy (Anti-PD-1)	Diet	FMD	Recruiting
NCT04316520	Metastatic renal cancer	20	Anti-PD-1 ± anti-CTLA-4	Diet	Ketogenic diet	Recruiting
NCT04957511	Gynecologic cancer	30	Immunotherapy	—	—	Recruiting
NCT04729322	Metastatic colorectal adenocarcinoma, Metastatic small intestinal adenocarcinoma	15	Anti-PD-1	FMT	FMT *via* colonoscopy and capsule	Recruiting
NCT04988841	Melanoma	60	Anti-CTLA-4 + anti-PD-1	Probiotics	MaaT013	Recruiting
NCT05094167	NSCLC	46	Anti-PD-1 + chemotherapy	Probiotics	V9	Recruiting
NCT05032014	Liver cancer	46	Anti-PD-1	Probiotics	M9	Recruiting
NCT04208958	Metastatic cancer, Melanoma, Gastric cancer, Gastroesophageal junction adenocarcinoma, Colorectal cancer	54	Anti-PD-1	Probiotics	VE800	Active, not recruiting
NCT02960282	Metastatic colorectal cancer	21	Chemotherapy or immunotherapy (Anti-PD-1/PD-L1)	—	—	Terminated (Slow accrual)
NCT05107427	Urothelial carcinoma	30	Anti-PD-1	Probiotics	MRx0518	Active, not recruiting
NCT04601402	NSCLC, Head and neck squamous cell carcinoma, Urothelial carcinoma	93	Anti-PD-1	Probiotics	GEN-001	Recruiting
NCT04264975	Solid tumor	60	Immunotherapy	FMT	FMT	Recruiting
NCT04521075	Metastatic melanoma, NSCLC	42	Anti-PD-1	FMT	FMT *via* capsules	Recruiting
NCT05083416	Head and neck cancer	52	Immunotherapy + chemotherapy	Diet	Prolonged nightly fasting	Recruiting

Targeting the microbiota to mitigate toxicities induced by ICIs is under active investigation. FMT has been demonstrated as a therapeutic modality for ICI-related colitis refractory to immunosuppressive therapies. Follow-up analysis revealed reconstitution of the gut microbiome after FMT treatment, with an inverse change in infiltrating levels between CD8^+^ T cells and Treg cells (Wang et al., 2018). At present, the Preventing Toxicity in Renal Cancer Patients Treated with Immunotherapy Using Fecal Microbiota Transplantation (PERFORM) study (NCT04163289) assessed the feasibility and safety of combining FMT with anti-PD-1 therapy in patients with metastatic RCC. The initial result observed an ORR of 44% (95% CI, 30–60), while 80% of patients (*n* = 8) experienced immune-related adverse events (irAEs), and four patients discontinued combination therapy due to irAEs. Further studies in larger cohorts are warranted (Fernandes et al., 2022). Additionally, FMT is being investigated for its safety and function in minimizing ICI-related toxicities in various cancers (Table 1).

## Diets

Dietary habits play a pivotal role in shaping microbiome variance among people, and their alterations can modify the gut microbiome, but not necessarily permanently. Dietary intervention targeting the gut microbiome has emerged as an appealing approach due to its safety and convenience, although it may have fewer striking effects than FMT or probiotics. Growing evidence supports the use of the ketogenic diet as an adjunctive strategy to the antitumor effects of ICI. In a murine melanoma model, the ketogenic diet and its main ketone body, three hydroxybutyrate (3HB), not only pronouncedly shifted the composition of the gut microbiota but also restored the effectiveness of ICI by inhibiting PD-L1 expression and promoting the expansion of CXCR3^+^ T cells (Ferrere et al., 2021). A clinical trial evaluating the potential effect of the ketogenic diet on immunotherapy outcomes is currently underway (NCT04316520).

The fasting mimicking diet (FMD), a plant-based, calorie-restricted, low-carbohydrate, low-protein diet, has been proposed as a potential anticancer dietary intervention to modulate gut microbiota composition and immune cell profiles. Recently, a clinical study focusing on the association of FMD intervention with ICIs in patients with NSCLC is ongoing (NCT03700437). Another phase II trial using metformin plus/minus cyclic FMD is currently being conducted in advanced LKB1-inactive lung cancer to increase the effectiveness of first line chemoimmunotherapy (NCT03709147). Patients with head and neck cancer are being studied to determine whether they will respond better to ICIs if they restrict their eating to an 8–10-h window each day and then fast for a more extended period each night (NCT05083416). Moreover, several clinical studies are evaluating the feasibility of high-fiber or low-protein diet and resistant starch in cancer patients who are receiving ICI treatment (Table 1). As mentioned above, there are some ongoing efforts to combine ICI with dietary intervention, but no results have been obtained thus far. It is necessary to further explore the potential mechanisms explaining the immunomodulatory effects of dietary intervention. Moreover, considering the limited influence of diets on gut microbiota, combining dietary intervention with FMT may help to expand the benefits of immunotherapy.

## Probiotics

Dietary interventions may appear to be easy to implement, but their impacts on the gut microbiota are often limited, and patients’ compliance is challenging to enforce and monitor. Direct administration of specific probiotics or their metabolites could be a superior choice due to their high efficiency but few side effects. Currently, numerous clinical studies are being conducted to further investigate the therapeutic potential of prebiotics in combination with immunotherapy due to their immunomodulatory effects on the host immune system (Table 1).

Various single strains of bacteria have been used as probiotics to improve clinical outcomes through modulation of the immune response. A preclinical study in syngeneic mouse models of breast and lung cancer demonstrated that the *E. gallinarum* strain MRX0518 could inhibit tumor growth in conjunction with an elevation in the CD8^+^ T cell: Treg ratio (Stevenson et al., 2018). Preliminary data from a small cohort of multiple cancer types demonstrated that oral live biotherapeutic MRx0518 enhanced antitumor activity, manifested by significant increases in genes and metagenes involved in antigen presentation, innate immune processes, interferon response, Th1 cells, and CD8^+^ cells (NCT03934827) (Lythgoe et al., 2021). The clinical studies of MRx0518 as a cotherapy with PD-1/PD-L1 inhibitors to treat various tumors are ongoing (NCT03637803, NCT05107427).

More interestingly, an increasing number of preclinical studies and clinical trials have reported that *Bifidobacteria* and *Lactic acid* bacteria can improve the curative effect of cancer immunotherapy. A single strain of *Bifidobacterium animals lactis* called EDP1503 has been shown to induce systemic antitumor immunity by elevating the production of cytokines (such as IFN- and CXCL10), activating NK cells and CD8^+^ T cells and triggering a proinflammatory signature within the TME (Gardner et al., 2019). The safety, tolerability, and efficacy of EDP1503 in combination with ICI have been evaluated in multiple cancer types (NCT03775850). EDP1503 administered with pembrolizumab was safe and well tolerated in patients with microsatellite stable colorectal cancer, without significant serious therapeutic toxicity (McHale et al., 2020). Again, the satisfactory safety and tolerability of EDP1503 and pembrolizumab combination therapy were proven in Triple-negative breast cancer (TNBC) patients, who experienced clinical benefit with an ORR of 18% and a disease control rate (DCR) of 27% in evaluable patients (*n* = 11) (Yuan et al., 2021). A phase 2 trial (NCT03595683) is presently recruiting patients with advanced melanoma to evaluate the efficacy of EDP1503 in enhancing the response to conventional immunotherapy.

A specific strain of *Lactobacillus lactis* known as GEN-001 can generate immunogenic metabolites, thus activating multiple immune cells, including T cells, DCs, and macrophages, potentially affecting the efficacy of immunotherapy (Chen et al., 2022). A phase I clinical study to investigate the safety, tolerability, and biological and clinical activities of GEN-001 in combination with avelumab in advanced solid tumor patients who have progressed during or after receiving ICI therapy is underway (NCT04601402). To explore the oral probiotics V9 (*Lactobacillus bifidobacterium*) and M9 (*Lactobacillus rhamnosus*) combined with PD-1 inhibitors for patients with NSCLC and liver cancer, two clinical studies were conducted (NCT05094167, NCT05032014).

CMB588 contains *Clostridium butyricum*, which belongs to SCFA-producing probiotics and has immunomodulatory activity. The addition of CBM588 prolonged median PFS (12.7 versus 2.5 months, hazard ratio (HR) 0.15, 95% CI 0.05–0.47, *p* < .001) and ORR (58% versus 20%) in patients with metastatic RCC receiving nivolumab plus ipilimumab. Furthermore, patients receiving CBM588 had alterations in their gut microbiota, including upregulation of rhamnose synthesis and increased SCFA propionate production (NCT03829111) (Dizman et al., 2022). Currently, many other clinical trials are underway to evaluate the safety and anticancer effect of commensal bacteria and immunotherapy combinations in various cancer patients (NCT04699721, NCT04167137, NCT04009122, NCT03817125).

Compared to FMT and single-strain probiotics, microbial ecosystem therapeutics (METs) are more effective and safer since they are composed of deliberately engineered bacterial communities derived from the intestinal microorganisms isolated from the feces of a healthy donor. Initial results from an early phase 1 study have thus far supported the safety of oral administration of MET-4 in combination with an anti-PD-1/PD-L1 inhibitor in cancer patients (NCT03686202) (Araujo et al., 2020). A consortium of 11 commensal strains isolated from the feces of healthy donors was found to improve the therapeutic efficacy of ICIs in mouse models by activating interferon-γ-producing CD8^+^ T cells in the intestine (Tanoue et al., 2019). This consortium, which is now being produced as a probiotic capsule called VE800, is undergoing Phase 1 clinical testing as an orally delivered therapy in combination with the anti-PD-1 inhibitor nivolumab (NCT04208958). MaaT013 is regarded as the next-generation FMT product with high standardization, produced from pooled healthy donors, and characterized by a highly consistent richness of 455 intestinal microbiome species. A prospective randomized clinical trial (NCT04988841) assessing the tolerance and clinical benefit of MaaT013 (administered *via* enema) in melanoma patients treated with CTLA-4 and PD1 inhibitors has begun. A phase 4 trial is now being conducted to assess the clinical efficacy of combination probiotics (Bifidobacterium, Lactobacillus, and Enterococcus capsules) in immunotherapy for patients with bladder urothelial cancer (NCT05220124).

### Microbes as biomarkers of clinical responses and adverse effects of checkpoint immunotherapy

Clear clinical evidence now reinforces the potential predictive capability of gut microbiota for ICI response and toxicities. To systematically assess gut microbiome features, the Predicting Response to Immunotherapy for Melanoma With Gut Microbiome and Metabolomics (PRIMM) (NCT03643289) study applied shotgun metagenomic sequencing on stool samples collected from five observational cohorts (*n* = 165) recruiting ICI-naive patients with advanced cutaneous melanoma who were planned to undergo anti-PD-1 therapy with or without anti-CTLA4 therapy. The researchers noted that the correlation between gut microbiota profiles and ICI responses is cohort dependent. When examining the microbiota in ICI respondents, it was found that a few taxa, notably *Bifidobacterium Pseudocatenulatum*, *Akkermansia muciniphila*, and two uncultivated species of *Roseburia*, were related to ORR and PFS (Lee et al., 2022). In addition, a large population-based trial (NCT04107168) intends to evaluate the potential of the microbiome as a biomarker of immunotherapy efficacy and toxicity in advanced cancer patients receiving different ICI therapies.

An observational clinical trial (NCT03688347) revealed multiple promising correlations between the gut microbiome and treatment response and the occurrence of irAEs in advanced lung cancer patients scheduled to undergo ICI-based treatment. Enrichment of *Bifidobacterium* (*p* = .001) and *Desulfovibrio* (*p* = .0002) was observed in the gut microbiome of patients without irAEs. In responders to combined chemoimmunotherapy, the relative abundance of *Clostridiales* (*p* = .018) was higher than that in non-responders, whereas *Rikenellaceae* (*p* = .016) was depleted in responders (Chau et al., 2021). There are also plenty of ongoing studies devoted to depicting the composition and/or changes of microbiomes and their products related to ICI responses and irAEs (NCT04204434, NCT04579978, NCT04189679, NCT04711330, NCT04954885, NCT03643289, NCT04957511, NCT02960282) in several solid cancers, including lung cancer, melanoma, gynecologic cancer, and colorectal cancer.

## Conclusion and future directions

Microbiota represents a rich source of small molecule drug discovery. However, emerging endeavors in microbiota mainly focus on manipulating microbiota directly to enhance immunotherapy efficacy. Small molecules derived from microbiota are rarely developed into drugs specific to immunotherapeutic effects. Identifying responsible effector molecules from microorganisms that affect host immunity and deciphering the corresponding regulatory mechanisms is a crucial step for the development of microbiota-based therapeutics. Moreover, multicenter trials are warranted to further evaluate the feasibility and safety of microbiota-based therapies in patients with cancer. As an increasing number of immunomodulators are discovered from microbiota, microbiota-derived medications will be developed and offer better options to improve the benefit of checkpoint immunotherapy.

## References

[B1] Allen-Vercoe E. Coburn B. (2020). A microbiota-derived metabolite augments cancer immunotherapy responses in mice. Cancer Cell 38 (4), 452–453. 10.1016/j.ccell.2020.09.005 32976777

[B2] Ansaldo E. Belkaid Y. (2021). How microbiota improve immunotherapy. Science 373 (6558), 966–967. 10.1126/science.abl3656 34446595

[B3] Araujo D. V. Oliva Bernal M. Tan T. J. Y. Heirali A. A. Schneeberger P. H. Pimentel Muniz T. (2020). First-in-class microbial ecosystem therapeutics 4 (MET4) in metastatic solid cancer patients treated with immunotherapy: MET4-IO. American Society of Clinical Oncology.

[B4] Baruch E. N. Youngster I. Ben-Betzalel G. Ortenberg R. Lahat A. Katz L. (2021). Fecal microbiota transplant promotes response in immunotherapy-refractory melanoma patients. Science 371 (6529), 602–609. 10.1126/science.abb5920 33303685

[B5] Baruch E. N. Youngster I. Ben-Betzalel G. Ortenberg R. Lahat A. Katz L. (2021). Fecal microbiota transplant promotes response in immunotherapy-refractory melanoma patients. Science 371 (6529), 602–609. 10.1126/science.abb5920 33303685

[B6] Botticelli A. Vernocchi P. Marini F. Quagliariello A. Cerbelli B. Reddel S. (2020). Gut metabolomics profiling of non-small cell lung cancer (NSCLC) patients under immunotherapy treatment. J. Transl. Med. 18 (1), 49. 10.1186/s12967-020-02231-0 32014010 PMC6998840

[B7] Buck M. D. Sowell R. T. Kaech S. M. Pearce E. L. (2017). Metabolic instruction of immunity. Cell 169 (4), 570–586. 10.1016/j.cell.2017.04.004 28475890 PMC5648021

[B8] Campbell C. McKenney P. T. Konstantinovsky D. Isaeva O. I. Schizas M. Verter J. (2020). Bacterial metabolism of bile acids promotes generation of peripheral regulatory T cells. Nature 581 (7809), 475–479. 10.1038/s41586-020-2193-0 32461639 PMC7540721

[B9] Chau J. Yadav M. Liu B. Furqan M. Dai Q. Shahi S. (2021). Prospective correlation between the patient microbiome with response to and development of immune-mediated adverse effects to immunotherapy in lung cancer. BMC cancer 21 (1), 808–814. 10.1186/s12885-021-08530-z 34256732 PMC8278634

[B10] Chen Y. Wu F-H. Wu P-Q. Xing H-Y. Ma T. (2022). The role of the tumor microbiome in tumor development and its treatment. Front. Immunol. 13, 935846. 10.3389/fimmu.2022.935846 35911695 PMC9334697

[B11] Connell E. Le Gall G. Pontifex M. G. Sami S. Cryan J. F. Clarke G. (2022). Microbial-derived metabolites as a risk factor of age-related cognitive decline and dementia. Mol. Neurodegener. 17 (1), 43. 10.1186/s13024-022-00548-6 35715821 PMC9204954

[B12] Coutzac C. Jouniaux J. M. Paci A. Schmidt J. Mallardo D. Seck A. (2020). Systemic short chain fatty acids limit antitumor effect of CTLA-4 blockade in hosts with cancer. Nat. Commun. 11 (1), 2168. 10.1038/s41467-020-16079-x 32358520 PMC7195489

[B13] Cryan J. F. O'Riordan K. J. Cowan C. S. M. Sandhu K. V. Bastiaanssen T. F. S. Boehme M. (2019). The microbiota-gut-brain Axis. Physiol. Rev. 99 (4), 1877–2013. 10.1152/physrev.00018.2018 31460832

[B14] Davar D. Dzutsev A. K. McCulloch J. A. Rodrigues R. R. Chauvin J-M. Morrison R. M. (2021). Fecal microbiota transplant overcomes resistance to anti–PD-1 therapy in melanoma patients. Science 371 (6529), 595–602. 10.1126/science.abf3363 33542131 PMC8097968

[B15] Devlin A. S. Fischbach M. A. (2015). A biosynthetic pathway for a prominent class of microbiota-derived bile acids. Nat. Chem. Biol. 11 (9), 685–690. 10.1038/nchembio.1864 26192599 PMC4543561

[B16] Dizman N. Meza L. Bergerot P. Alcantara M. Dorff T. Lyou Y. (2022). Nivolumab plus ipilimumab with or without live bacterial supplementation in metastatic renal cell carcinoma: A randomized phase 1 trial. Nat. Med. 28 (4), 704–712. 10.1038/s41591-022-01694-6 35228755 PMC9018425

[B17] Fernandes R. Parvathy S. N. Ernst D. S. Haeryfar M. Burton J. Silverman M. (2022). Preventing adverse events in patients with renal cell carcinoma treated with doublet immunotherapy using fecal microbiota transplantation (FMT): Initial results from perform a phase I study. American Society of Clinical Oncology.

[B18] Ferrere G. Alou M. T. Liu P. Goubet A-G. Fidelle M. Kepp O. (2021). Ketogenic diet and ketone bodies enhance the anticancer effects of PD-1 blockade. JCI insight 6 (2), e145207. 10.1172/jci.insight.145207 33320838 PMC7934884

[B19] Finlay B. B. Goldszmid R. Honda K. Trinchieri G. Wargo J. Zitvogel L. (2020). Can we harness the microbiota to enhance the efficacy of cancer immunotherapy? Nat. Rev. Immunol. 20 (9), 522–528. 10.1038/s41577-020-0374-6 32661409

[B20] Furusawa Y. Obata Y. Fukuda S. Endo T. A. Nakato G. Takahashi D. (2013). Commensal microbe-derived butyrate induces the differentiation of colonic regulatory T cells. Nature 504 (7480), 446–450. 10.1038/nature12721 24226770

[B21] Gardner H. A. Kashyap S. Ponichtera H. Sandy P. Parameswaran P. Carlson M. (2019). Monoclonal microbial EDP1503 to induce antitumor responses via gut-mediated activation of both innate and adaptive immunity. J. Clin. Oncol. 37 (15), e14241. 10.1200/jco.2019.37.15_suppl.e14241

[B22] Goc J. Sonnenberg G. F. (2022). Harnessing microbiota to improve immunotherapy for gastrointestinal cancers. Cancer Immunol. Res. 10 (11), 1292–1298. 10.1158/2326-6066.CIR-22-0164 36166399 PMC10424780

[B23] Hang S. Paik D. Yao L. Kim E. Trinath J. Lu J. (2019). Bile acid metabolites control T(H)17 and T(reg) cell differentiation. Nature 576 (7785), 143–148. 10.1038/s41586-019-1785-z 31776512 PMC6949019

[B24] He B. Hoang T. K. Wang T. Ferris M. Taylor C. M. Tian X. (2017). Resetting microbiota by Lactobacillus reuteri inhibits T reg deficiency-induced autoimmunity via adenosine A2A receptors. J. Exp. Med. 214 (1), 107–123. 10.1084/jem.20160961 27994068 PMC5206500

[B25] Huang J. Jiang Z. Wang Y. Fan X. Cai J. Yao X. (2020). Modulation of gut microbiota to overcome resistance to immune checkpoint blockade in cancer immunotherapy. Curr. Opin. Pharmacol. 54, 1–10. 10.1016/j.coph.2020.06.004 32619934

[B26] Huang J. Liu D. Wang Y. Liu L. Li J. Yuan J. (2022). Ginseng polysaccharides alter the gut microbiota and kynurenine/tryptophan ratio, potentiating the antitumour effect of antiprogrammed cell death 1/programmed cell death ligand 1 (anti-PD-1/PD-L1) immunotherapy. Gut 71 (4), 734–745. 10.1136/gutjnl-2020-321031 34006584 PMC8921579

[B27] Huttenhower C. Kostic A. D. Xavier R. J. (2014). Inflammatory bowel disease as a model for translating the microbiome. Immunity 40 (6), 843–854. 10.1016/j.immuni.2014.05.013 24950204 PMC4135443

[B28] Inamura K. (2020). Roles of microbiota in response to cancer immunotherapy. Semin. Cancer Biol. 65, 164–175. 10.1016/j.semcancer.2019.12.026 31911189

[B29] Karayama M. Masuda J. Mori K. Yasui H. Hozumi H. Suzuki Y. (2021). Comprehensive assessment of multiple tryptophan metabolites as potential biomarkers for immune checkpoint inhibitors in patients with non-small cell lung cancer. Clin. Transl. Oncol. 23 (2), 418–423. 10.1007/s12094-020-02421-8 32533317 PMC7854397

[B30] Kim C. H. (2021). Control of lymphocyte functions by gut microbiota-derived short-chain fatty acids. Cell. Mol. Immunol. 18 (5), 1161–1171. 10.1038/s41423-020-00625-0 33850311 PMC8093302

[B31] Kim S. Covington A. Pamer E. G. (2017). The intestinal microbiota: Antibiotics, colonization resistance, and enteric pathogens. Immunol. Rev. 279 (1), 90–105. 10.1111/imr.12563 28856737 PMC6026851

[B32] Kostic A. D. Chun E. Robertson L. Glickman J. N. Gallini C. A. Michaud M. (2013). Fusobacterium nucleatum potentiates intestinal tumorigenesis and modulates the tumor-immune microenvironment. Cell Host Microbe 14 (2), 207–215. 10.1016/j.chom.2013.07.007 23954159 PMC3772512

[B33] Lee K. A. Thomas A. M. Bolte L. A. Björk J. R. de Ruijter L. K. Armanini F. (2022). Cross-cohort gut microbiome associations with immune checkpoint inhibitor response in advanced melanoma. Nat. Med. 28 (3), 535–544. 10.1038/s41591-022-01695-5 35228751 PMC8938272

[B34] Levy M. Blacher E. Elinav E. (2017). Microbiome, metabolites and host immunity. Curr. Opin. Microbiol. 35, 8–15. 10.1016/j.mib.2016.10.003 27883933

[B35] Li H. Bullock K. Gurjao C. Braun D. Shukla S. A. Bossé D. (2019). Metabolomic adaptations and correlates of survival to immune checkpoint blockade. Nat. Commun. 10 (1), 4346. 10.1038/s41467-019-12361-9 31554815 PMC6761178

[B36] Li M. van Esch B. Wagenaar G. T. M. Garssen J. Folkerts G. Henricks P. A. J. (2018). Pro- and anti-inflammatory effects of short chain fatty acids on immune and endothelial cells. Eur. J. Pharmacol. 831, 52–59. 10.1016/j.ejphar.2018.05.003 29750914

[B37] Li W. Hang S. Fang Y. Bae S. Zhang Y. Zhang M. (2021). A bacterial bile acid metabolite modulates T(reg) activity through the nuclear hormone receptor NR4A1. Cell Host Microbe 29 (9), 1366–1377.e9. e9. 10.1016/j.chom.2021.07.013 34416161 PMC9064000

[B38] Loo T. M. Kamachi F. Watanabe Y. Yoshimoto S. Kanda H. Arai Y. (2017). Gut microbiota promotes obesity-associated liver cancer through PGE(2)-mediated suppression of antitumor immunity. Cancer Discov. 7 (5), 522–538. 10.1158/2159-8290.CD-16-0932 28202625

[B39] Lythgoe M. Adriani M. Stebbing J. Clark J. Pickford E. Frampton A. (2021). 543P Neoadjuvant MRx0518 treatment is associated with significant gene and metagene signature changes in solid tumours. Ann. Oncol. 32, S607. 10.1016/j.annonc.2021.08.1065

[B40] Mager L. F. Burkhard R. Pett N. Cooke N. C. A. Brown K. Ramay H. (2020). Microbiome-derived inosine modulates response to checkpoint inhibitor immunotherapy. Science 369 (6510), 1481–1489. 10.1126/science.abc3421 32792462

[B41] Matson V. Chervin C. S. Gajewski T. F. (2021). Cancer and the microbiome-influence of the commensal microbiota on cancer, immune responses, and immunotherapy. Gastroenterology 160 (2), 600–613. 10.1053/j.gastro.2020.11.041 33253684 PMC8409239

[B42] McHale D. Francisco-Anderson L. Sandy P. Shariffudin S. Goldberg M. Gardner H. (2020). P-325 Oral delivery of a single microbial strain, EDP1503, induces anti-tumor responses via gut-mediated activation of both innate and adaptive immunity. Ann. Oncol. 31, S195. 10.1016/j.annonc.2020.04.407

[B43] Miller W. H. Routy B. Jamal R. Ernst D. S. Logan D. Esfahani K. (2022). Fecal microbiota transplantation followed by anti–PD-1 treatment in patients with advanced melanoma. American Society of Clinical Oncology.

[B44] Nomura M. Nagatomo R. Doi K. Shimizu J. Baba K. Saito T. (2020). Association of short-chain fatty acids in the gut microbiome with clinical response to treatment with nivolumab or pembrolizumab in patients with solid cancer tumors. JAMA Netw. Open 3 (4), e202895. 10.1001/jamanetworkopen.2020.2895 32297948 PMC7163404

[B45] Pham F. Moinard-Butot F. Coutzac C. Chaput N. (2021). Cancer and immunotherapy: A role for microbiota composition. Eur. J. Cancer 155, 145–154. 10.1016/j.ejca.2021.06.051 34375896

[B46] Proietti E. Rossini S. Grohmann U. Mondanelli G. (2020). Polyamines and kynurenines at the intersection of immune modulation. Trends Immunol. 41 (11), 1037–1050. 10.1016/j.it.2020.09.007 33055013

[B47] Ridlon J. M. Harris S. C. Bhowmik S. Kang D. J. Hylemon P. B. (2016). Consequences of bile salt biotransformations by intestinal bacteria. Gut Microbes 7 (1), 22–39. 10.1080/19490976.2015.1127483 26939849 PMC4856454

[B48] Scher J. U. Sczesnak A. Longman R. S. Segata N. Ubeda C. Bielski C. (2013). Expansion of intestinal Prevotella copri correlates with enhanced susceptibility to arthritis. Elife 2, e01202. 10.7554/eLife.01202 24192039 PMC3816614

[B49] Schroeder B. O. Bäckhed F. (2016). Signals from the gut microbiota to distant organs in physiology and disease. Nat. Med. 22 (10), 1079–1089. 10.1038/nm.4185 27711063

[B50] Seton-Rogers S. (2021). Microbiota links to immunotherapy toxicity. Nat. Rev. Cancer 21 (9), 540. 10.1038/s41568-021-00390-w 34282322

[B51] Singh R. P. Bashir H. Kumar R. (2021). Emerging role of microbiota in immunomodulation and cancer immunotherapy. Semin. Cancer Biol. 70, 37–52. 10.1016/j.semcancer.2020.06.008 32580024

[B52] Stevenson A. Panzica A. Holt A. Laute Caly D. Ettore A. Delday M. (2018). “Host-microbe interactions mediating antitumorigenic effects of MRX0518, a gut microbiota-derived bacterial strain,” in breast, renal and lung carcinoma (American Society of Clinical Oncology).

[B53] Sun L. Cai J. Gonzalez F. J. (2021). The role of farnesoid X receptor in metabolic diseases, and gastrointestinal and liver cancer. Nat. Rev. Gastroenterol. Hepatol. 18 (5), 335–347. 10.1038/s41575-020-00404-2 33568795

[B54] Sun M. Ma N. He T. Johnston L. J. Ma X. (2020). Tryptophan (Trp) modulates gut homeostasis via aryl hydrocarbon receptor (AhR). Crit. Rev. Food Sci. Nutr. 60 (10), 1760–1768. 10.1080/10408398.2019.1598334 30924357

[B55] Tanoue T. Morita S. Plichta D. R. Skelly A. N. Suda W. Sugiura Y. (2019). A defined commensal consortium elicits CD8 T cells and anti-cancer immunity. Nature 565 (7741), 600–605. 10.1038/s41586-019-0878-z 30675064

[B56] Trompette A. Gollwitzer E. S. Pattaroni C. Lopez-Mejia I. C. Riva E. Pernot J. (2018). Dietary fiber confers protection against flu by shaping Ly6c(-) patrolling monocyte hematopoiesis and CD8(+) T cell metabolism. Immunity 48 (5), 992–1005. e8. 10.1016/j.immuni.2018.04.022 29768180

[B57] Vezza T. Algieri F. Garrido-Mesa J. Utrilla M. P. Rodríguez-Cabezas M. E. Baños A. (2019). The immunomodulatory properties of propyl-propane thiosulfonate contribute to its intestinal anti-inflammatory effect in experimental colitis. Mol. Nutr. Food Res. 63 (5), e1800653. 10.1002/mnfr.201800653 30516875

[B58] Wang T. Gnanaprakasam J. N. R. Chen X. Kang S. Xu X. Sun H. (2020). Inosine is an alternative carbon source for CD8(+)-T-cell function under glucose restriction. Nat. Metab. 2 (7), 635–647. 10.1038/s42255-020-0219-4 32694789 PMC7371628

[B59] Wang Y. Wiesnoski D. H. Helmink B. A. Gopalakrishnan V. Choi K. DuPont H. L. (2018). Fecal microbiota transplantation for refractory immune checkpoint inhibitor-associated colitis. Nat. Med. 24 (12), 1804–1808. 10.1038/s41591-018-0238-9 30420754 PMC6322556

[B60] Yuan Y. Lee J. S. Yost S. E. Frankel P. H. Ruel C. Egelston C. A. (2021). A phase II clinical trial of pembrolizumab and enobosarm in patients with androgen receptor-positive metastatic triple-negative breast cancer. Oncologist 26 (2), 99–e217. 10.1002/onco.13583 33141975 PMC7873338

[B61] Zhou C. B. Fang J. Y. (2018). The regulation of host cellular and gut microbial metabolism in the development and prevention of colorectal cancer. Crit. Rev. Microbiol. 44 (4), 436–454. 10.1080/1040841X.2018.1425671 29359994

[B62] Zhou C. B. Zhou Y. L. Fang J. Y. (2021). Gut microbiota in cancer immune response and immunotherapy. Trends Cancer 7 (7), 647–660. 10.1016/j.trecan.2021.01.010 33674230

